# Postoperative Hiatal Hernia after Ivor Lewis Esophagectomy—A Growing Problem in the Age of Minimally Invasive Surgery

**DOI:** 10.3390/jcm12175724

**Published:** 2023-09-01

**Authors:** Jasmina Kuvendjiska, Robert Jasinski, Julian Hipp, Mira Fink, Stefan Fichtner-Feigl, Markus K. Diener, Jens Hoeppner

**Affiliations:** 1 Department of General and Visceral Surgery, University Medical Center, 79106 Freiburg, Germany; 2 Faculty of Medicine, Albert-Ludwigs-University of Freiburg, 79085 Freiburg, Germany; 3 Department of Surgery, University Medical Center Schleswig-Holstein, 23538 Lübeck, Germany

**Keywords:** esophagectomy, esophageal cancer, hiatal hernia, minimally invasive surgery, postoperative complication

## Abstract

Background: Even though minimally invasive esophagectomy is a safe and oncologically effective procedure, several authors have reported an increased risk of postoperative hiatal hernia (PHH). This study evaluates the incidence and risk factors of PHH after hybrid minimally invasive (HMIE) versus open esophagectomy (OE). Methods: A retrospective single-center analysis was performed on patients who underwent Ivor Lewis esophagectomy between January 2009 and April 2018. Computed tomography scans and patient files were reviewed to identify the PHH. Results: 306 patients were included (152 HMIE; 154 OE). Of these, 23 patients (8%) developed PHH. Most patients (13/23, 57%) were asymptomatic at the time of diagnosis and only 4 patients (17%) presented in an emergency setting with incarceration. The rate of PHH was significantly higher after HMIE compared to OE (13.8% vs. 1.3%, *p* < 0.001). No other risk factors for the development of PHH were identified in uni- or multi-variate analysis. Surgical repair of PHH was performed in 19/23 patients (83%). The recurrence rate of PHH after surgical repair was 32% (6/19 patients). Conclusions: The development of PHH is a relevant complication after hybrid minimally invasive esophagectomy. Although most patients are asymptomatic, surgical repair is recommended to avoid incarceration with potentially fatal outcomes. Innovative techniques for the prevention and repair of PHH are urgently needed.

## 1. Introduction

Esophageal carcinoma is the eighth most common cancer in the world and the sixth most common cause of cancer death worldwide [1]. Although multimodal treatment protocols are progressively applied, surgery remains the central part of curative treatment for most patients. Different techniques of esophagectomy with variations in terms of resection and reconstruction are performed internationally. Established techniques include a transhiatal, two-field transthoracic (Ivor Lewis) and tri-incisional resection (abdominal incision, right thoracotomy and left neck incision.). The two-field Ivor Lewis procedure is the technique most used in the Western world today. It includes an abdominal part as well as a thoracic part of the resection via right thoracotomy. The reconstruction is performed with intrathoracic anastomosis. For most patients, a gastric sleeve is used for reconstruction and esophageal replacement, whereas reconstruction with jejunum or colon is far less commonly performed. Esophagectomy is associated with significant surgical morbidity. Postoperative morbidity has been reduced by the use of minimally invasive techniques, regardless of the respective technique, e.g., complete minimally invasive or hybrid techniques [2,3,4]. The main benefit of minimally invasive esophagectomy is a reduction in pulmonary morbidity [2,3]. Since the oncological outcome remains comparable to open esophagectomy, totally and hybrid minimally invasive esophagectomy has become the standard procedure.

At the Medical Centre of the University of Freiburg, hybrid minimally invasive esophagectomy (HMIE) and open esophagectomy (OE) are both applied as highly standardized procedures [5,6]. Over the years, HMIE has become the gold standard technique for esophageal resections in our center. However, with the consecutive clinical implementation of HMIE as the standard technique, an increased occurrence of postoperative paraconduit hiatal hernia (PHH) has been observed. The occurrence of PHH after totally minimally invasive esophagectomy (TMIE) was recently reported to be more frequent than after open esophagectomy [7,8,9]. Although there are several studies on the occurrence of PHH after a TMIE procedure, there are very few studies on the HMIE procedure. Although PHHs are often asymptomatic, there is a risk of ischemia of the herniated intestine, with potentially fatal consequences [10].

Overall, the literature on PHH is still quite limited, including mostly retrospective studies and two meta-analyses based on retrospective data. A further problem in the existing literature on this topic is the frequent use of heterogeneous patient collectives with diverse operating techniques such as gastrectomy, transhiatal esophagectomy, Ivor Lewis or McKeown esophagectomy. However, research on the comparison between HMIE and OE in terms of PHH occurrence is still limited.

This study aims to determine and compare the incidence, potential risk factors and outcome of PHH after OE and HMIE.

## 2. Materials and Methods

### 2.1. Study Design

We conducted a single-center retrospective study with the objective of determining incidence and outcomes of PHH after esophagectomy. We included patients operated between January 2009 and April 2018. The included patients underwent either a fully open Ivor Lewis esophagectomy (OE) or hybrid minimally invasive esophagectomy (HMIE). The applied technique of HMIE included a laparoscopic abdominal and open thoracic part via right thoracotomy as described in prior reports [11,12]. All patients were reconstructed using a gastric sleeve, and patients with other forms of reconstruction were excluded from the analysis. In both groups, a widening of the hiatus by intraoperative hiatotomy of the right crura and prophylactic colopexy of the transverse colon to the abdominal wall were sometimes performed based on the surgeon’s preference. Clinical follow-up of all patients was conducted routinely in our specialized outpatient department for Upper GI Surgery. Occurrence of PHH was identified by a computed tomography (CT) performed in all patients either during the regular oncological follow-up or due to specific symptoms. PHH was defined as herniation of abdominal organs (excluding the gastric conduit) through the esophageal hiatus. Anatomical details about PHH were identified through evaluation of CT scans. Patient data, including medical history, disease symptoms and management of the hiatal hernia, were extracted from patient charts. The study was approved by the Medical Ethics Committee of the University of Freiburg (File No. 253/19).

### 2.2. Statistical Analysis

Descriptive statistics were applied for the patient characteristics and postoperative complications. Univariate analysis was performed to assess the role of potential risk factors for the occurrence of PHH. All parameters with a significance of *p* < 0.1 were subsequently entered into a binary logistic regression analysis with backwards stepwise variable selection. A statistical significance level of 0.05 was used and statistical analysis was performed using IBM SPSS Statistics version 28.0 (IBM Corp, Armonk, NY, USA).

## 3. Results

### 3.1. Study Population

**Figure 1:**
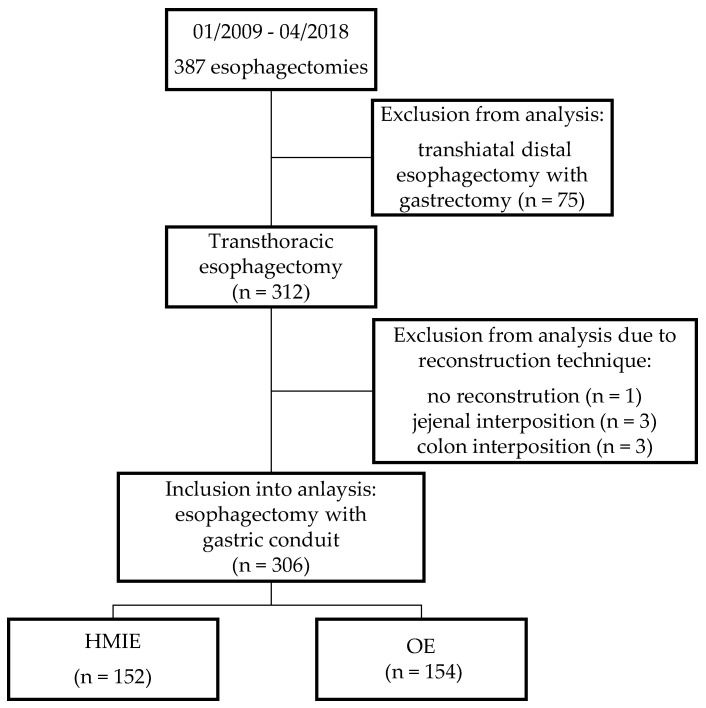
Flowchart of the study population selection. OE = open esophagectomy, HMIE = hybrid minimally invasive esophagectomy.

Overall, 387 patients underwent an esophagectomy between January 2009 and April 2018. Seventy-four of these patients underwent a transhiatal distal esophagectomy with gastrectomy and were excluded from the analysis. One patient underwent an emergency esophagectomy without reconstruction and a further six patients received an esophagectomy with a reconstruction other than gastric pull-up (colonic interposition (*n* = 3); jejunal interposition (*n* = 3)), and were also excluded in the analysis. Finally, 306 patients who underwent esophagectomy and reconstruction with gastric conduit were included. HMIE was performed in 152 patients (49.67%) and OE in 154 patients (50.3%) (Figure 1). There were no conversions to open surgery in the HMIE group. Overall, 295 procedures were performed for esophageal cancer, with esophageal adenocarcinoma being the most frequent diagnosis (*n* = 215, 70.3%). Most of the patients received neoadjuvant treatment in the form of chemoradiation (*n* = 104, 34%) or chemotherapy alone (*n* = 140, 45.6%), in accordance with national guidelines. Demographic data are shown in Table 1.

### 3.2. Postoperative Hiatal Hernia

**Figure 2:**
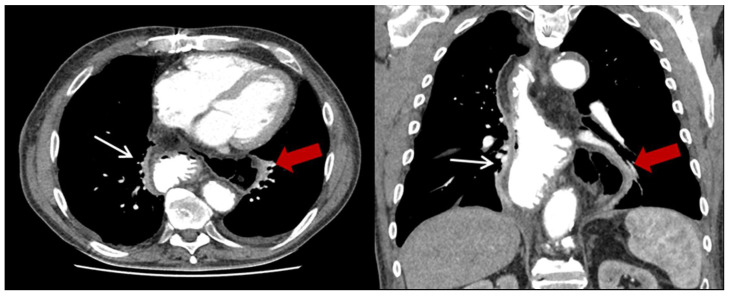
Exemplary CT-scan of a hiatal hernia on the left thoracic side after HMIE. Gastric conduit marked with thin white arrow; herniated colon marked with thick red arrow.

**Figure 3:**
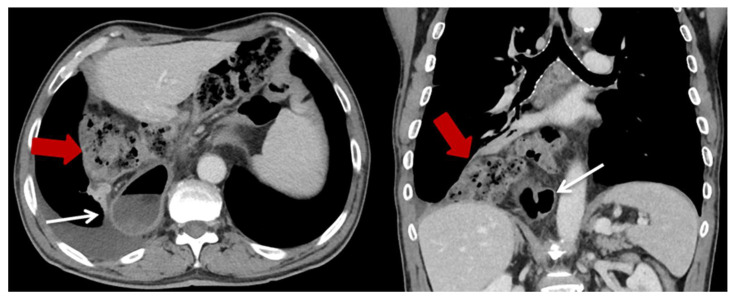
Exemplary CT-scan of a hiatal hernia on the right thoracic side after HMIE. Gastric conduit marked with thin white arrow; herniated colon marked with thick red arrow.

Overall, 23 of the patients (7.5%) developed PHH in the postoperative course. The median follow-up time was 21 months. The occurrence of PHH was significantly increased after HMIE (*n* = 21, 13.8%) compared to OE (*n* = 2, 1.3%, *p* < 0.001). The median time to diagnosis after HMIE was 14 months, and it was 75.5 months after OE. In every analyzed case, the transverse colon was the herniated organ, with additional herniation of the small intestine in three cases. Most frequently, the hiatal hernia occurred on the left thoracic side (*n* = 18, 78.3%) (Figure 2), followed by small mediastinal hernias in the middle (*n* = 3, 13%). In one case, the hernia occurred on the right side (4.4%) (Figure 3) and in another case bilaterally (4.4%).

Univariate analysis was performed to assess the role of potential risk factors for the occurrence of PHH. The examined factors included patient characteristics, surgical technique factors and postoperative complications. The following parameters were included in the univariate analysis: age, gender, ASA, operating technique (HMIE vs. OE), T and N stadium, intraoperative hiatotomy, prophylactic colopexy, preexisting hiatal hernia, BMI, nicotine abuse, COPD, CHD, diabetes mellitus, use and type of neoadjuvant treatment, postoperative pneumonia, postoperative mediastinitis, anastomotic leakage, anastomotic stricture and delayed gastric emptying (Table 2). Five parameters showed a significance *p* < 0.1 and were entered into a binary logistic regression analysis. Using backwards stepwise variable selection, the following variables were excluded from the equation: prophylactic colopexy, intraoperative hiatotomy, T-stage and N-stage, leaving HMIE as the only significant factor related to an increased development of PHH after esophagectomy (Table 3).

**Figure 4:**
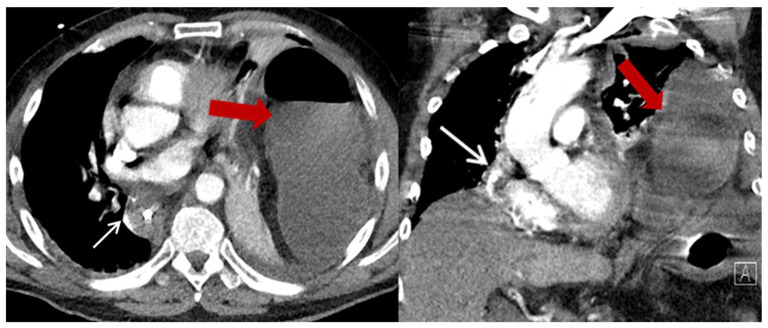
Exemplary CT-scan of an incarcerated hiatal hernia on the left thoracic side with a clear mediastinal shift to the right. Gastric conduit marked with thin white arrow; herniated colon marked with thick red arrow.

In most cases, PHH were asymptomatic (*n* = 13, 56.5%) and were diagnosed coincidentally by CT scans performed as part of the routine oncologic follow-up (Table 4). The remaining patients (*n* = 10, 43.5%) were symptomatic, with four patients (17.4%) presenting as an emergency (one ileus and three incarcerations of intestine) (Figure 4). The most common symptom was abdominal pain and discomfort. Surgical repair was strongly recommended to every patient with diagnosed PHH. Out of 23 patients, 4 patients declined surgical intervention despite the strong recommendation. Meanwhile, 19 patients underwent surgical repair (82.6%), of which 4 (21%) were in emergency settings. One of the patients operated in an emergency setting died in the ICU due to septic shock.

**Figure 5:**
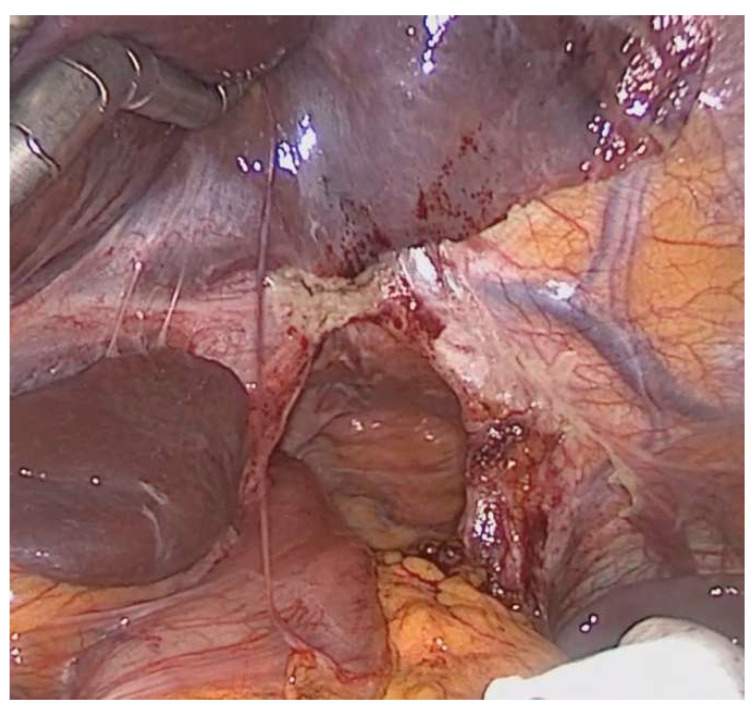
Exemplary picture of extremely enlarged, scarified and inflexible hiatus after HMIE. The herniated intestine (colon) has already been removed from the thorax.

The operation on the PHH was usually performed laparoscopically (*n* = 16, 84.2%) with only two conversions to open surgery (Table 4). The standard procedure involved a repositioning of the intestine and colopexy. In 42%, a gastropexy to the hiatus was performed to minimize the hiatal opening. If feasible, a suture hiatoplasty was also performed (47.4%). Since this is not always possible due to wide and scarified hiatus (Figure 5), different procedures were used like mesh augmentation of the hiatus (21%) or a curtain-like suspension of the mesocolon in front of the hiatus by a wide colopexy to the abdominal wall (31.6%). We observed a significant recurrence of the hiatal hernia after repair (*n* = 6, 31.6%). Here, again, the transverse colon was always involved, with additional herniation of small intestine in one case. The surgical repair of the hernia was repeated in five of the cases. One patient did not undergo surgical treatment due to tumor progression.

## 4. Discussion

HMIE with gastric pull-up is performed with high safety as a standardized operative technique for patients with esophageal cancer, as reported previously [5]. Reduction in the intraoperative and postoperative complications can be achieved by the use of minimally invasive techniques like HMIE or TMIE [2,3,4]. Despite the great advantages over the years with the implementation of minimally invasive techniques in esophageal surgery, one complication seemed to increase: the PHH [13]. The literature on this growing problem is limited since only retrospective studies and two meta-analyses based on retrospective data [13,14,15,16,17] are available. Most of the studies focus on totally minimally invasive esophagectomy or use a heterogeneous patient collective with diverse operating techniques such as gastrectomy, transhiatal distal esophagectomy, Ivor Lewis or McKeown esophagectomy (Table 5).

In the present study, we investigated the occurrence of PHH after an Ivor Lewis esophagectomy performed in the period from January 2009 to April 2018. The patient collective involved only patients with Ivor Lewis esophagectomies performed either as open or hybrid minimally invasive. We observed a significantly higher rate of hiatal hernia after HMIE in comparison with OE (13.8% vs. 1.3%, *p* < 0.001). The transverse colon was herniated in every case of PHH and 78.3% of the PHHs were on the left thoracic side, similar to the study results of Brenkman et al. [27].

A recently published meta-analysis reports 2.6% PHHs after OE and 6.3% after TMIE including Ivor Lewis, McKeown and transhiatal esophagectomies [15]. Regarding HMIE, 6.7% of PHHs were reported based only on two studies [15,18,25]. The mentioned studies report different outcomes. While Vallböhmer et al. report a low incidence of 2.7% PHHs after HMIE, the incidence reported by Mathews et al. estimates 10% PHHs after HMIE [18,25]. Here, we have to take into account that the operating technique used in the study by Vallböhmer et al. was a two-stage HMIE consisting of a laparoscopic mobilization of the stomach followed by a second operation (open transthoracic esophagectomy) with a mean delay of 4 days [18]. In a mixed collective of 414 patients operated, including different techniques of Ivor Lewis esophagectomy, Pucceti et al. report 5.4% PHHs after HMIE but no significant association between minimally invasive surgery and the occurrence of PHH [8].

The suspected mechanism for the higher incidence of PHH after HMIE or TMIE is the presence of less adhesion after minimally invasive surgery, which allows a higher mobility of the intestine. Also, it is suggested that the surgical widening of the hiatus is larger in minimally invasive techniques. However, in our study, there was no significant impact of a hiatotomy on the development of PHH. Regarding the extension of the widening of the hiatus, a comparison between the groups was not possible due to the retrospective nature of our study.

Although most patients are asymptomatic, surgical repair is recommended since a significant percentage of patients develop unpredictable incarceration of the herniated intestine. This can easily lead to perforation of the intestine with consecutive mediastinitis, septic shock and high morbidity and mortality. In order to prevent such potentially fatal complications, we recommend elective surgical repair regardless of whether the patient is symptomatic or not. Besides the minimally invasive operating technique, no other risk factors could be identified for the development of PHH. This should not discourage the use of minimally invasive techniques, since their advantages have already been proven [2,3]. Nevertheless, this new risk factor for postoperative complications must raise awareness in postoperative follow-up. Since a large part of the patients do not report any clear symptoms, care must be given in the process of reviewing the follow-up imaging, to not miss possible PHH. Furthermore, the occurrence of PHH after HMIE demands further refinement of the technique. Until now, no prophylactic measures during esophagectomy have been shown to significantly reduce the occurrence of PHH. In our study, there was also no significant impact of prophylactic colopexy of the transverse colon to the abdominal wall performed during the esophagectomy in terms of PHH.

A further problem regarding the treatment of PHH is the high recurrence rate after surgical repair. We observed a PHH recurrence in 31.6% of the operated patients. Price et al. analyzed over 2000 patients with PHH after MIE who underwent a hiatoplasty with or without mesh reinforcement and reported morbidity rates of up to 60% and recurrence rates of 13.3%, similar to the recently published data by Oppelt et al. [20,32]. Kent et al. also analyzed the PHH repair with or without the use of mesh and reported recurrence rates of up to 29%, which is comparable to our results [19]. The main reason for the high recurrence rates is probably the technical difficulty of covering/closing the often extremely wide hiatal opening and thinned crura. Depending on the large hiatal opening, it is sometimes not possible to approximate the crura and an alternative solution for the closure of the gap may be needed, such as the use of Goretex mesh or ligamentum teres hepatis, as previously reported by our group [33]. Regardless of the technique used, maximum care must be taken not to damage the feeding gastroepiploic vessels of the conduit.

The current study has several limitations, including the retrospective design. Particularly technical details, such as hiatal widening during operations, cannot be evaluated in a retrospective setting. Furthermore, some learning curve bias cannot be excluded due to the implementation of HMIE during the course of the study. Also, since there is no standard treatment for PHH, the surgical treatment chosen was based on the surgeon’s preferences. Loss to follow-up is minimized due to the close routine oncological follow-up in our outpatient setting.

Prospective studies are needed to examine possible prophylactic measures during the esophagectomy such as cruroplasty or wide colopexy, in order to reduce the occurrence of PHH. Furthermore, in the absence of a standardized repair technique for PHH, only prospective randomized studies comprising comparisons of different techniques and mesh grafts can help us find the optimal repair solution in the case of PHH and reduce recurrences.

## 5. Conclusions

PHH is a relevant complication after HMIE, with frequent herniation of the transverse colon in the left thoracic side. Even though it is mostly asymptomatic, PHH can lead to incarceration of the intestine with a potentially fatal outcome and should undergo surgical repair. Surgeons must be aware of this complication and remain vigilant in the postoperative radiological follow-up. Further refinement of the minimally invasive esophagectomy technique as well as the PHH repair technique is needed to reduce the occurrence of PHH and recurrence after repair.

## Figures and Tables

**Table 1:** Clinicopathological characteristics of the study population.

	HMIE	OE
Patients (*n*)	152	154
Male gender (*n*, %)	117 (77%)	133 (86.4%)
Age (median, years)	62	63
BMI (median, kg/m^2^)	26.5	25
ASA-score (*n*, %)		
I	1 (0.7%)	2 (1.3%)
II	75 (49.3%)	76 (49.4%)
III	72 (47.4%)	73 (47.4%)
IV	4 (2.6%)	3 (1.9%)
Tumor histology (*n*, %)		
Adenocarcinoma	114 (75%)	101 (65.6%)
Squamouscell carcinoma	33 (21.7%)	47 (30.5%)
Others	5 (3.3%)	6 (3.9%)
Neoadjuvant treatment (*n*, %)		
None	34 (22.4%)	27 (17.7%)
Chemotherapy	68 (44.7%)	72 (46.7%)
Chemoradiation	49 (32.2%)	55 (35.7%)
Radiotherapy	1	0
Comorbidity (*n*, %)		
Nicotine abuse	57 (37.5%)	67 (43.5%)
CHD	16 (10.5%)	18 (11.7%)
Diabetes	20 (13.2%)	21 (13.6%)
Adipositas	25 (16.4%)	21 (13.6%)
COPD	22 (14.5%)	19 (12.3%)

BMI = body mass index; ASA = American Society of Anaesthesiologists; CHD = coronary heart disease; COPD = chronic obstructive pulmonary disease.

**Table 2:** Univariate analysis of risk factors for postoperative hiatal hernia.

Variable	Hiatal Hernia (*n*, %)	No Hiatal Hernia (*n*, %)	*p*-Value
Patients	23	283	
Male gender	16 (69.6%)	234 (82.7%)	0.118
Age (years)			0.759
<65	12 (52.2%)	157 (55.5%)	
≥65	11 (47.8%)	126 (44.5%)	
BMI (kg/m^2^)			0.66
<25	9 (39.1%)	124 (43.8%)	
≥25	14 (60.9%)	159 (56.2%)	
ASA-score			0.29
I + II	14 (60.9%)	140 (49.5%)	
III + IV	9 (39.1%)	143 (50.5%)	
Comorbidity			
Nicotine abuse	10 (43.5%)	114 (40.3%)	0.76
CHD	2 (8.7%)	32 (11.3%)	0.7
Diabetes	2 (8.7%)	39 (13.8%)	0.49
COPD	3 (13%)	38 (13.4%)	0.96
Preexisting hiatal hernia	2 (8.7%)	30 (10.6%)	0.78
T stadium			0.046
T 0–2	20 (87%)	183 (66.8%)	
T 3–4	3 (13%)	91 (33.2%)	
N- stadium			0.1
N0	18 (78.3%)	166 (61%)	
N+	5 (21.7%)	106 (39%)	
Neoadjuvant treatment			
Chemotherapy	18 (78.3%)	226 (79.9%)	0.86
Radiochemotherapy	8 (34.8%)	97 (34.3%)	0.96
Operation technique			
HMIE	21 (91.3%)	131 (46.3%)	<0.001
OE	2 (8.7%)	152 (53.7%)	
Hiatotomy	20 (87%)	196 (69.3%)	0.073
Colopexy	19 (82.6%)	171 (60.4%)	0.035
Postoperative complications			
Pneumonia	6 (26.1%)	62 (21.9%)	0.64
Mediastinitis	0	6 (2.1%)	0.48
Anastomotic leakage	1 (4.3%)	17 (6%)	0.75
Anastomotic stenosis	3 (13%)	15 (5.3%)	0.13
Delayed gastric emptying	2 (8.7%)	28 (9.9%)	0.85

BMI = body mass index; ASA = American Society of Anaesthesiologists; CHD = coronary heart disease; COPD = chronic obstructive pulmonary disease, OE = open esophagectomy, HMIE = hybrid minimally invasive esophagectomy.

**Table 3:** Multivariate analysis of risk factors for postoperative hiatal hernia.

Variable		Odds Ratio	95%-CI	*p*-Value
HMIE vs. OE	OE	Reference	Reference	<0.001
	HMIE	11.812	2.717–51.366	

**Table 4:** Characteristics of the hiatal hernia and surgical repair technique.

Characteristics	HH (*n* = 23) (%)
Content of HH, *n* (%)	
Colon	23 (100)
Additionally small bowel	3 (13)
Position of the HH *n* (%)	
Left thoracic side	18 (78.3)
Right thoracic side	1 (4.4)
Both sides	1 (4.4)
Lower mediastinum	3 (13)
Symptoms *n* (%)	
None	13 (56.5)
Abdominal pain and discomfort	10 (43.5)
Ileus/incarceration	4 (17.4)
Surgical repair, *n* (%)	
Laparoscopic	16 (84.2)
Conversion	2 (12.5)
Open	3 (15.8)
Gastropexy	8 (42)
Hiatoplasty	9 (47.4)
Mesh augmentation	4 (21)
Colopexy	15 (78.9)
Recurrence rate, *n* (%)	6 (31.6)

**Table 5:** Literature review on post-esophagectomy PHH.

Study	Year	Patients (*n*)	Included Operations	PHH Overall (%)	PHH OE (%)	PHH TMIE (%)	PHH HMIE (%)	PHH Recurrence (%)
Vallböhmer et al. [18]	2007	355	OE Ivor Lewis HMIE (two-stage)	2.5	2.4	/	2.7	/
Kent et al. [19]	2008	1075	OE and TMIE Ivor Lewis OE and TMIE McKeown	4	0.8	2.8	/	29
Price et al. [20]	2011	2182	OE Ivor Lewis OE transhiatal	0.7	0.7	/	/	13.3
Ganeshan et al. [21]	2013	440	OE Ivor Lewis	15	12		/	44
			OE McKeown		17			
			OE transhiatal		24			
			TMIE			10		
Bronson et al. [13]	2014	114	TMIE Transhiatal TMIE McKeown	8	/	8	/	12.5
Benjamin et al. [22]	2015	120	MIE	5.8	/	5.8	/	20
Messenger et al. [23]	2015	273	OE, TMIE and HMIE Ivor Lewis	4	1	13.2		18
Severino et al. [24]	2016	390	HMIE	8.2	/	/	8.2	19
Matthews et al. [25]	2016	631	OE and TMIE Ivor Lewis OE and TMIE McKeown HMIE Gastrectomy	5.5	2	7	10	26
Andreou et al. [26]	2017	471	OE Ivor Lewis	2.8	2.7	/	/	N/A
			Open gastrectomy		0.7			
			Open extended gastrectomy		6.1			
Brenkman et al. [27]	2017	657	OE and TMIE Ivor Lewis	7	4	10	/	15
			OE and TMIE McKeown		4	7	/	
			OE and TMIE transhiatal		11	4	/	
Gooszen et al. [28]	2018	851	OE and TMIE Ivor Lewis	2.5	0	9.4	/	19
			OE and TMIE McKeown		1.4	1.6		
			OE and TMIE transhiatal		1.3	2.3		
Gust et al. [9]	2019		OE, HMIE or TMIE Ivor Lewis OE or TMIE McKeown OE and MIE transhiatal	1.2	0.7	1.4		/
Takeda et al. [29]	2019	328	TMIE McKeown HMIE McKeown RAMIE McKeown	2.4	/	2.4		0
Iwasaki et al. [16]	2020	113	TMIE McKeown	9.7	/	9.7	/	/
Fuchs et al. [30]	2020		HMIE Ivor Lewis		/	/	N/A.	7.7
Hanna et al. [17]	2020	258	OE and MIE Ivor Lewis, transhiatal and McKeown	31	N/A	N/A	N/A	17
Lubbers et al. [10]	2021	307	TMIE Ivor Lewis TMIE McKeown	2.6	/	2.6	/	38
Puccetti et al. [8]	2021	414	OE, TMIE and HMIE Ivor Lewis	5.3	2.9	8.3	5.4	13.6
Chung et al. [31]	2021	49	MIE	14	/	14	/	80

PHH = paraconduit hiatal hernia, OE = open esophagectomy, TMIE = totally minimally invasive esophagectomy, MIE = minimally invasive esophagectomy, HMIE = hybrid minimally invasive esophagectomy, N/A = not applicable.

## Data Availability

The data presented in this study are available on request from the corresponding author.
